# Restorative treatment decisions for carious lesions: Do Russian dentists and dental students apply minimal intervention dentistry?

**DOI:** 10.1186/s12903-021-01978-2

**Published:** 2021-12-15

**Authors:** Sergei N. Drachev, Alexandra S. Galieva, Tatiana N. Yushmanova, Elena A. Polivanaya, Lina Stangvaltaite-Mouhat, Rania Al-Mahdi, Jukka Leinonen, Linda Maria Stein, Nadezhda G. Davidova, Mohammed Al-Haroni

**Affiliations:** 1 Department of Clinical Dentistry, Faculty of Health Sciences, UiT The Arctic University of Norway, N-9037 Tromsø, Norway; 2 Department of Prosthodontics, Dental Faculty, Northern State Medical University, Arkhangelsk, Russia; 3 Department of Therapeutic Dentistry, Dental Faculty, Northern State Medical University, Arkhangelsk, Russia; 4 Oral Health Centre of Expertise in Eastern Norway, Oslo, Norway

**Keywords:** Carious lesion, Minimal intervention dentistry, Restorative treatment decision, Russia, Northern State Medical University

## Abstract

**Background:**

The concept of minimal intervention dentistry (MID) includes both delayed restorative treatment and conservative caries removal, and is now recognised as an evidence-based approach for dental caries management. In order to determine if dental professionals in Russia are incorporating this concept into their clinical practice, we investigated the restorative treatment decisions of Russian dentists and dental students, and the factors associated with these decisions.

**Methods:**

We included 171 general dental practitioners and dental therapists (collectively referred to here as “dentists”) from North-West Russia, and 76 dental undergraduate students from the Northern State Medical University in Arkhangelsk (response rate of 11.5% and 67.9%, respectively). Participants completed a questionnaire, which collected background information (sex, region of work, place of dental school graduation, practice type, years of working experience, working in an urban or rural area, and specialisation in restorative dentistry) and information on restorative treatment decisions for proximal and occlusal carious lesions of permanent teeth. Treatment options in accordance with MID were defined as intervention at dentin level and minimally invasive cavity preparation. Multinomial logistic regression was used for statistical analysis.

**Results:**

For the proximal carious lesion, 9.4% of participants said they would employ both MID treatment options; 60.7% said they would choose only one; and 29.9% said they would use neither option. For the occlusal carious lesion, the corresponding figures were 37.2%, 52.1%, and 10.7%. No differences in restorative treatment options were observed among general dental practitioners, dental therapists, and dental students. For the proximal carious lesion, dentists from regions outside Arkhangelsk had 4.15 (95% confidence interval [CI] 1.13–15.27) times higher odds of following one versus both MID treatment options. For the occlusal carious lesion, working experience above 15 years was associated with higher odds of using only one versus both MID treatment options (adjusted odds ratio = 3.04, 95% CI 1.33–6.91). Almost all respondents preferred tooth-coloured materials for restorations; more than 75% chose resin-based composite.

**Conclusions:**

The majority of Russian dentists and dental students do not apply the MID concept when treating dental caries in permanent teeth. Clinical protocols on dental caries treatment and dental school curriculums should be updated to place an enhanced focus on evidence-based practice and preventive strategies. Further studies with larger samples of Russian dentists and dental students and alternative methods of recruitment are needed to validate our results.

## Background

Dental caries is a common non-communicable health condition worldwide, and it can impact a person’s functional, social, and psychological well-being, as well as cause considerable economic and quality of life burdens [1, 2]. However, dental caries is also highly preventable [3], and recommended intervention strategies should primarily be either non-invasive, such as reduced sugar consumption, regular tooth-brushing, and topical application of fluoride; or micro-invasive, such as sealing and infiltration [4]. Nevertheless, absence or failure of these measures will lead to a need for restorative treatment involving removal of carious tissue and restoration placement [4, 5]. Criteria for restorative treatment have changed significantly since the 1970s: the initiation of restorative treatment in the early stages of the carious process and the removal of all carious tissue until caries-resistant areas are reached are no longer supported [4, 5]. Instead, the concept of minimal intervention dentistry (MID) has been introduced in order to keep teeth healthy and functional as long as possible [5–8]. MID focuses on early caries detection and risk assessment, non-invasive/micro-invasive interventions to prevent, arrest, and reverse carious lesions, and conservative caries removal when restorative treatment is indicated [7]. The study of treatment strategies is important to determine the extent to which dental professionals apply the modern, patient-centred, evidence-based MID concept in the management of dental caries.

Espelid et al. developed a questionnaire to explore dental professionals’ restorative treatment decisions. It asks about the thresholds for initiating restorative treatment, cavity preparation, and restorative materials that respondents would use for the proximal and occlusal carious lesions of permanent teeth [9, 10]. The questionnaire was first used among Scandinavian dental professionals and later in countries like the USA [11], France [12], Spain [13], Croatia [14], and Kuwait [15]. The studies showed a wide variation in restorative treatment decisions in different countries [9–15]. Although an apparent increase in the use of resin-based composite was found, no significant temporal worldwide trend was observed, neither for threshold for initiating restorative treatment nor for cavity preparation [5, 16]. Along with inconsistencies in clinical guidelines for dental professionals, researchers attributed this to differences in dental education, as dental schools may not introduce evidence-based teaching to dental undergraduate students or incorporate MID in cariology curriculums [8, 17].

To our knowledge, there is no information on restorative treatment decisions among dentists in Russia, where there is a high prevalence of dental caries in children [18] and young adults [19]. In one study, filled teeth accounted for 90.2% of the sum of decayed, missing, filled teeth (DMFT index) in medical and dental students in North-West Russia. The study also showed that DMFT index increased among students who reported regular dental visits [19]. These findings may suggest that Russian dentists focus on restorative treatment instead of non-invasive/micro-invasive interventions. In Russia, general dental practitioners and specialists in restorative dentistry (hereafter referred to as “dental therapists”) perform most restorative treatment of permanent teeth in adults. General dental practitioners must complete 5 years of university studies and pass an accreditation exam. Dental therapists must complete an additional 2 years of clinical residency (or additional specialisation of 4 months for those who graduated university before 2016 and completed their clinical internship).

In order to determine if dental professionals in Russia are incorporating MID into their clinical practice, we investigated the restorative treatment decisions of Russian dentists and dental undergraduate students, and the factors associated with these decisions.

## Methods

### Study setting, population, and sample size calculation

The study area included the European North-West regions of Russia: the Arkhangelsk, Murmansk, and Vologda Regions; the Karelia and Komi Republics; and the Nenets Autonomous Okrug. All general dental practitioners and dental therapists from these regions were eligible for the survey. Dental professionals from other fields (prosthetics, oral surgery, paediatric dentistry, and orthodontics) were not invited to participate. According to the Federal State Statistics Service, a total of 2128 dental professionals of all specialties were registered in the chosen regions in 2018 [20], but to our knowledge, information on the specific number of general dental practitioners and dental therapists (referred to collectively hereafter as “dentists”) was not available. We assumed this proportion to be ~ 70% of all dental professionals, and thus considered that the general population of the present survey consisted of 1490 dentists.

There is only one dental school in the study area: the Northern State Medical University (NSMU) located in the city of Arkhangelsk. Dental students in their fifth (i.e., final) year of education at the NSMU were also eligible to participate, of whom there were 112 (76.8% females) during the study period (2020–2021 academic year).

Taking into consideration an outcome prevalence of 50%, a confidence level of 95%, and error margin of 5%, the minimum sample size was calculated as 306 dentists and 87 dental students. Assuming a low response rate, we invited all dentists that could be reached in the study area, and all fifth-year dental students at the NSMU.

### Sampling

The survey was conducted from October 2020 to January 2021. An online questionnaire was developed using the electronic questionnaire tool “Nettskjema”, provided by the University of Oslo, Norway, and several strategies were used to recruit dentists. One strategy was to send e-mail invitations to regional chief dentists in the study area. The invitations included a study link, which led to a website that included information on the survey, the informed consent form, and the online questionnaire. The regional chief dentists were asked to forward the invitation to other dentists in their region on our behalf. Another strategy was to invite dentists through social media. Dentists attending continuous education courses at the NSMU during the data collection period were also informed about the survey during their courses and invited to participate. Finally, snowball sampling was used: participating dentists were asked to forward the e-mail invitation to any eligible colleagues. If a dentist wanted to participate, but could not complete the electronic study documents, he/she could contact the principal investigator (S. N. D.) or the Dean of Dental Faculty of NSMU (N.G.D.) to obtain paper copies. The completed paper copies could be left in the Dean’s office at any time during the data collection period. Fifth-year dental students at the NSMU were informed about the survey verbally and invited to participate during a scheduled curriculum lecture. A total of 171 dentists (response rate of 11.5%; Table 1) and 76 dental students (response rate of 67.9%) agreed to participate, signed an informed consent form, completed the questionnaire, and were included in statistical analysis.

**Table 1:** Total number of dentists and number who participated in the survey by regions, n (%)

Region	Total number of dentists in 2018^a^	Number included in the survey
Arkhangelsk	512 (34.3)	103 (60.2)
Vologda	292 (19.6)	24 (14.0)
Komi Republic	274 (18.4)	18 (10.5)
Murmansk	265 (17.8)	18 (10.5)
Republic of Karelia	132 (8.9)	7 (4.1)
Nenets Autonomous Okrug	15 (1.0)	1 (0.6)
Total	1490 (100)	171 (100)

^a^Dentists include general dental practitioners and dental therapists; the total number of dentists was calculated as ~ 70% of the total number of all dental professionals that was obtained from Federal State Statistics Service, by regions [20]

### Questionnaire

We used the questionnaire originally developed by Espelid et al. [9, 10]. The English version was translated into Russian using a forward–backward translation technique. Before the survey began, the Russian version of the questionnaire was pilot tested on 11 fourth-year dental students at the NSMU and four dentists who did not participate in the actual survey.

All questions on restorative treatment decisions for the proximal and occlusal carious lesions in the questionnaire refer to a fictional 20-year-old patient who visits a dentist annually, has good oral hygiene and low caries activity, and uses fluoride toothpaste. Questions 1 to 3 covered proximal carious lesions. In question 1, six schematic radiographic grades of proximal caries progress from outer one-half of enamel (grade 1) to inner one-third of dentin (grade 6) were shown on the distal surface of an upper second premolar [9], and participants were asked to choose the earliest grade at which immediate restorative treatment would be indicated. Responses were categorised as early intervention at enamel level (grades 1–3) and delayed intervention at dentin level (grades 4–6) [21]. Question 2 covered cavity preparation, with three possible responses: (1) traditional Class II preparation, (2) tunnel preparation, and (3) saucer-shaped preparation. Responses 2 and 3 were categorised as minimally invasive cavity preparation. In question 3, respondents were asked to choose one of nine materials for restoring the proximal carious lesion: (1) resin-based composite, (2) glass ionomer cement, (3) resin-modified glass ionomer cement, (4) sandwich technique: glass ionomer cement and composite, (5) dental amalgam, (6) compomer, (7) ceramic inlay, (8) gold inlay, and (9) other material.

Questions 4 to 6 focused on occlusal carious lesions. Question 4 presented five photographs of different clinical appearances of occlusal caries progress in a lower second molar [10]: (1) white or brown discolouration in enamel, no cavitation detected clinically, no radiographic signs of caries; (2) small cavity formation, or discolouration of the fissure with a surrounding opaque or grey zone of enamel and/or no radiographic evidence of caries; (3) moderately sized cavity and/or radiolucency in the outer 1/3 of the dentin; (4) large cavity and/or radiolucency in the middle 1/3 of the dentin; (5) extensive cavity and/or radiolucency in the inner 1/3 of the dentin. The respondents were asked to state the earliest grade at which immediate restorative treatment would be indicated. Responses were dichotomised as early intervention at enamel level (grades 1–2) and delayed intervention at dentin level (grades 3–5). Question 5 covered preparation technique, with three possible responses: (1) preparation limited to carious area, (2) preparation of the entire occlusal fissure system, (3) other type of preparation (for ex., preparation for an inlay). Responses 1 and 2 were considered minimally invasive and traditional cavity preparation, respectively. In question 6, as in question 3, respondents were asked to choose one of nine materials for restoring the occlusal carious lesion.

To assess the criterion validity of the instrument, participants were asked to express their opinion in relation to the following statement: “Faced with an incipient active carious lesion one should always perform a restoration technique based on cavity preparation” [13]. The response options included (1) strongly agree, (2) agree, (3) disagree, (4) strongly disagree. Respondents who agreed or strongly agreed with the statement had higher odds of replying that they would intervene at enamel level than at dentin level for the proximal carious lesion (odds ratio [OR] = 2.60, 95% confidence interval [CI] 1.33–5.11, *p* = 0.005) and the occlusal carious lesion (OR = 2.38, 95% CI 1.32–4.29, *p* = 0.004), which provided evidence of criterion validity.

The questionnaire also collected background information on dentists’ sex, region of work (Arkhangelsk Region, other), place of dental school graduation (NSMU, other), practice type (public, private, both), years of working experience (< 5, 5–9, 10–14, 15–19, ≥ 20), working on urban or rural area, and specialisation in restorative dentistry (yes, dental therapist; no, general dental practitioner). For dental students, only sex was considered.

### Statistical analysis

Descriptive statistics were used to define frequency distributions in restorative treatment decisions and background characteristics. To measure associations regarding grades at which participants reported that restorative treatment was indicated for the proximal and occlusal carious lesions, the Spearman’s rho correlation coefficient was applied. The chi-square test was used to compare the proportion of dentists and dental students who chose different cavity preparations. Binary logistic regression analysis was performed to measure the association between restorative treatment decisions for the proximal and occlusal carious lesions. To examine factors associated with restorative treatment decisions, multinomial logistic regression models were constructed separately for the proximal and occlusal carious lesions, with the dependent variable coded as 0 = intervention at dentin level and using minimally invasive cavity preparation (reference category), 1 = intervention at enamel level and using traditional cavity preparation, 2 = intervention at dentin level and using traditional cavity preparation or intervention at enamel level and using minimally invasive cavity preparation. Background characteristics (study group, sex, region of work, place of dental school graduation, dental practice type, working experience, and working in urban or rural area) were used as the independent variables. Independent variables with a level of significance (*p*) < 0.2 in the bivariable analysis were entered in multivariable multinomial logistic regression model. The regression analysis results are presented as ORs with 95% CIs. Statistical analysis was performed using IBM SPSS Statistics for Macintosh version 25.0 (IBM Corp., Armonk, New York, USA). All statistical tests were two-tailed, and the level of significance (*p*) was set at 0.05.

## Results

A total of 171 dentists and 76 dental students were included in the statistical analysis. One hundred and forty-two dentists (83.0%) and all dental students completed the electronic questionnaire, whereas 29 dentists (17.0%) preferred to fill in the paper questionnaire. The majority of the respondents were women, who represented 80.1% and 77.6% of dentists and dental students, respectively. Ninety percent of the dentists graduated from the NSMU and reported working in an urban area. Nearly half of the dentists worked in public dental practices, had working experience of less than 5 years, and had no specialisation in restorative dentistry, i.e., were general dental practitioners (Table 2).

**Table 2:** Characteristics of the sample of Russian dentists, n = 171

Characteristics	n (%)
Sex	
Male	34 (19.9)
Female	137 (80.1)
Place of dental school graduation	
Northern State Medical University in Arkhangelsk	153 (89.5)
Other	18 (10.5)
Dental practice type	
Public	77 (45.0)
Private	54 (31.6)
Both public and private	40 (23.4)
Working experience, years	
Less than 5	78 (45.6)
5–9	26 (15.2)
10–14	16 (9.4)
15–19	17 (9.9)
More than 20	34 (19.9)
Working in urban or rural area	
Urban	158 (92.4)
Rural	13 (7.6)
Specialization in restorative dentistry	
Yes (dental therapist)	97 (56.7)
No (general dental practitioner)	74 (43.3)

**Fig. 1:**
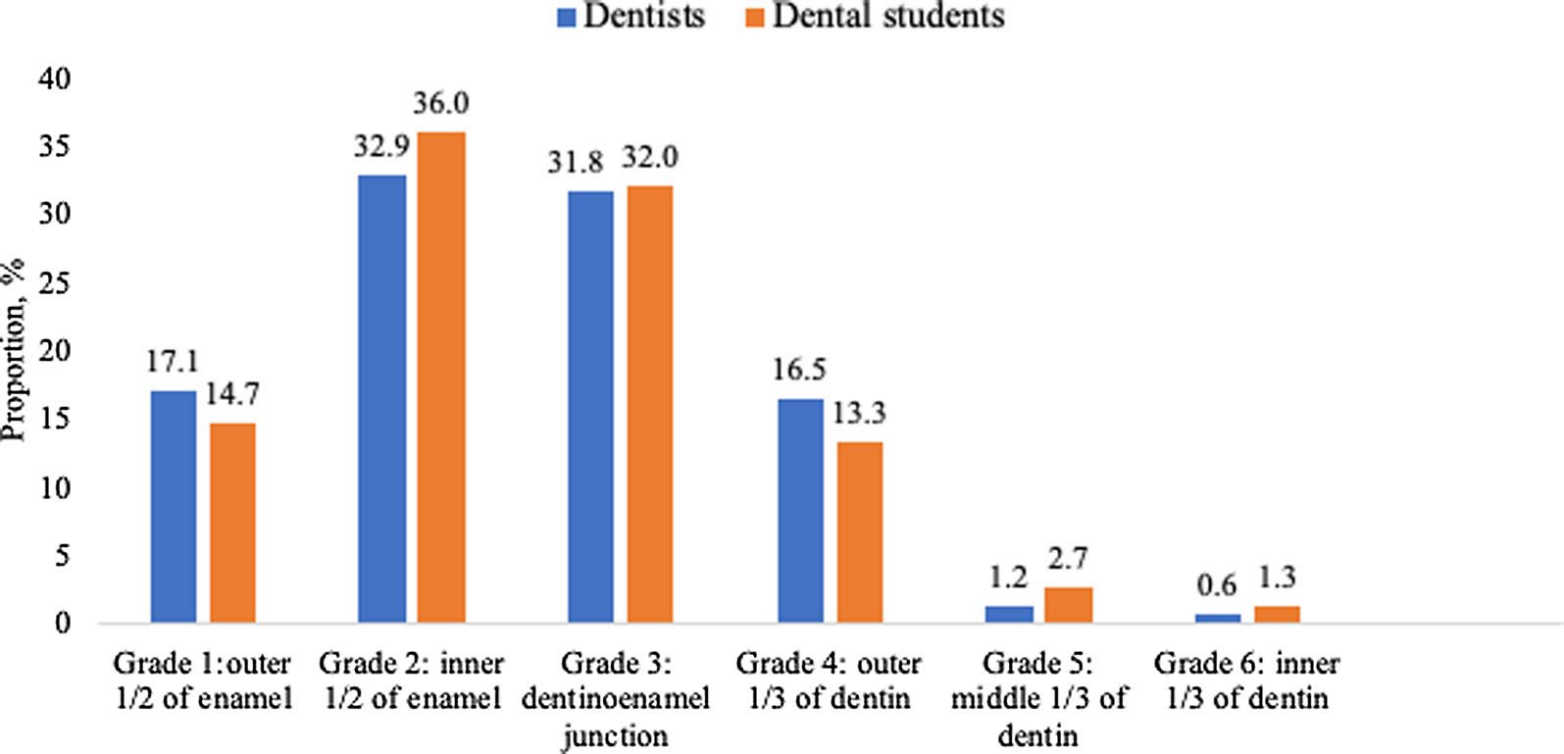
Threshold used by Russian dentists (n = 170) and dental students (n = 75) for initiating restorative treatment of the proximal carious lesion

Half of the respondents answered that immediate restorative treatment was indicated when the proximal carious lesion was confined to enamel (grades 1 and 2). One-third replied that it was indicated when the lesion reached the dentinoenamel junction, but not further (grade 3). Altogether, one out of six respondents said that restorative treatment was indicated at the level of outer one-third of dentin (grade 4), and only a few said that such treatment was indicated only when the lesion extended to the middle one-third of the dentin or deeper (grades 5 and 6). Similar results were found in dentists and dental students (Fig. 1).

Of the 171 dentists, 60.2% preferred to use minimally invasive cavity preparation for the proximal carious lesion (tunnel and saucer-shaped preparation, 21.6% and 38.6%, respectively); 39.8% chose traditional Class II preparation. Similar results were observed among dental students: of the 75 who answered this question, 24.0%, 41.3%, and 34.7% said they would use tunnel, saucer-shaped, and traditional Class II preparation, respectively (χ^2^ = 0.584, df = 2, *p* = 0.747). Ninety-nine percent of dentists and all dental students reported that they would choose tooth-coloured materials to restore the proximal carious lesion.

**Fig. 2:**
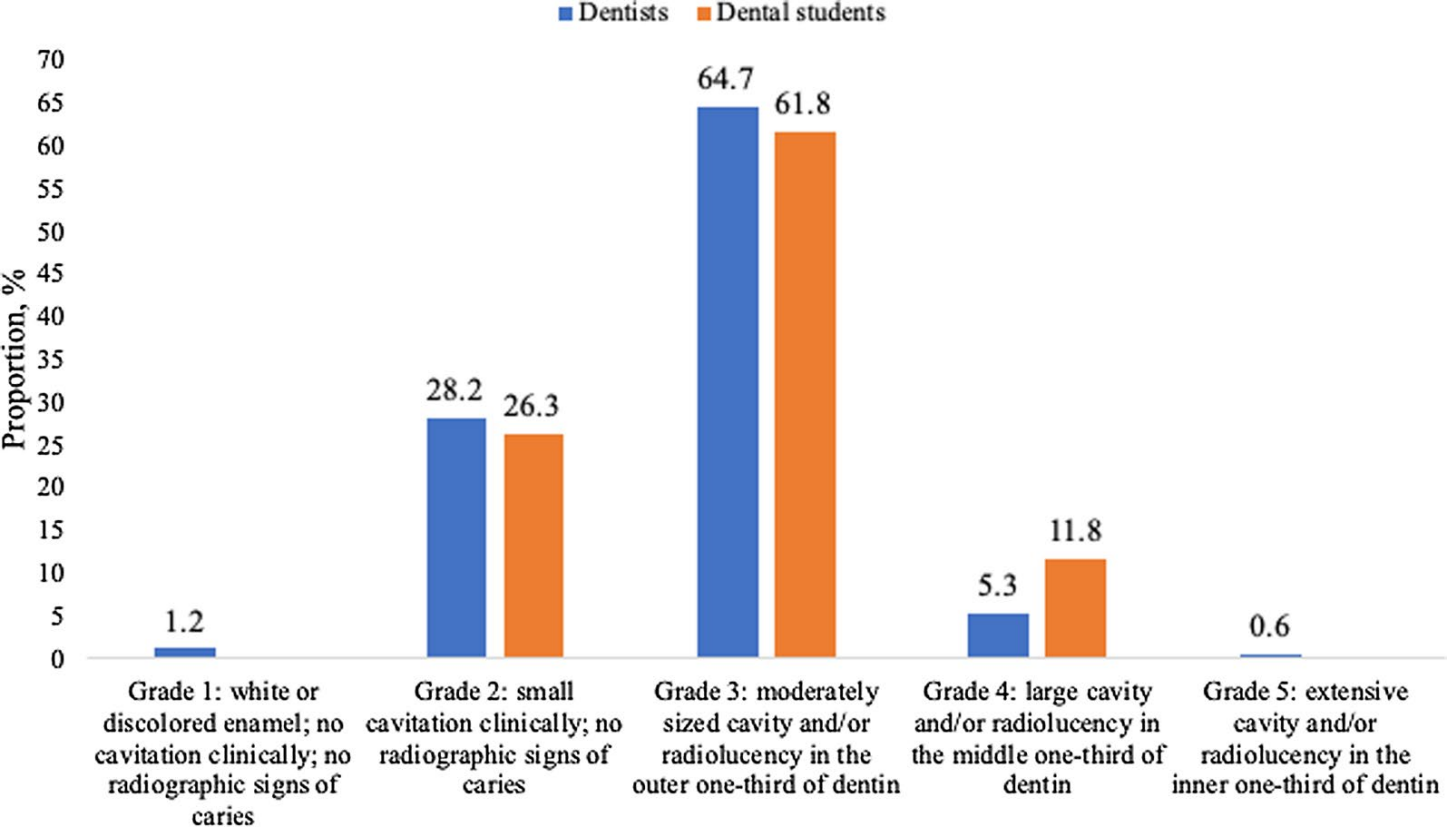
Threshold used by Russian dentists (n = 170) and dental students (n = 76) for initiating restorative treatment of the occlusal carious lesion

Close to a third of the participants considered that immediate restorative treatment was indicated when the initial occlusal carious lesion was confined to enamel (grades 1 and 2). Nearly two-thirds said this was indicated when the lesion reached the outer one-third of dentin (grade 3). Only one out of thirteen respondents said restorative treatment was only indicated when the lesion reached the middle or inner one-third of dentin (grades 4 and 5). Similar results were observed in dentists and dental students (Fig. 2).

There was a significant correlation between the proximal and the occlusal carious lesions regarding the grade at which immediate restorative treatment was reported to be indicated, both in dentists (Spearman’s rho = 0.384, *p* < 0.001) and in dental students (Spearman’s rho = 0.420, *p* < 0.001). Moreover, respondents who said that restoration was indicated at the enamel level of the proximal carious lesion (grades 1–3) were more likely to say the same thing about the occlusal carious lesion (grades 1–2, OR = 6.68, 95% CI 2.00–22.38, *p* = 0.002).

Of the 170 dentists who answered the question on cavity preparation for the occlusal carious lesion, 51.8% preferred a preparation with the removal of the carious part of fissure; 47.6% would prepare the entire occlusal fissure system, and 0.6% chose preparation for an inlay. Among dental students (n = 76), the corresponding figures were 57.9%, 38.2%, and 3.9%, respectively. No significant differences were found between the proportions of dentists and dental students who would prepare the carious part of fissure versus the entire occlusal fissure system (χ^2^ = 1.384, df = 1, *p* = 0.239). There was no association between minimally invasive cavity preparation for the proximal and occlusal carious lesions neither in dentists (OR = 1.63, 95% CI 0.87–3.03, *p* = 0.125) nor in dental students (OR = 1.39, 95% CI 0.52–3.73, *p* = 0.517).

Ninety-eight percent of dentists and all dental students reported that they would choose tooth-coloured materials to restore the occlusal carious lesion. Resin-based composite was found to be the restorative material of choice, both for the proximal and occlusal carious lesions (Table 3).

**Table 3:** Dental materials preferred by Russian dentists and dental students for restoring the proximal and occlusal carious lesions

Dental material	Proximal carious lesion, n (%)		Occlusal carious lesion, n (%)	
	Dentists, n = 166	Dental students, n = 76	Dentists, n = 168	Dental students, n = 76
Resin-based composite	126 (75.9)	58 (76.3)	133 (79.2)	66 (86.8)
GIC	4 (2.4)	3 (3.9)	3 (1.8)	2 (2.6)
Resin-modified GIC	6 (3.6)	4 (5.3)	6 (3.6)	3 (3.9)
Combination of GIC and composite	24 (14.5)	7 (9.2)	14 (8.3)	4 (5.3)
Compomer	4 (2.4)	3 (3.9)	8 (4.8)	–
Gold inlay	2 (1.2)	–	1 (0.6)	–
Ceramic inlay	–	1 (1.3)	–	1 (1.3)
Dental amalgam	–	–	3 (1.8)	–

*GIC* Glass ionomer cement

For the proximal carious lesion, 9.4% of participants chose both of the MID treatment options: intervention at dentin level and minimally invasive cavity preparation; 60.7% chose only one MID treatment option; and 29.9% did not choose either. For the occlusal carious lesion, corresponding figures were 37.2%, 52.1%, and 10.7%. The results of multinomial logistic regression analysis that explored factors associated with restorative treatment decisions are presented in Tables 4 and 5. For the proximal carious lesion, dentists practicing outside the Arkhangelsk Region had 4.15 (95% CI 1.13–15.27) times higher odds to use only one versus both MID treatment options. No significant differences in restorative treatment decisions for the proximal carious lesion were observed between categories of other variables considered (Table 4). For the occlusal carious lesion, working experience above 15 years was significantly associated with higher odds of using only one versus both MID treatment options (Table 5). This association remained significant after adjustment for region of work, practice type, and working in a rural or urban area (OR = 3.04, 95% CI 1.33–6.91, *p* = 0.008).

**Table 4:** Associations between restorative treatment decisions for the proximal carious lesion and selected variables among Russian dentists and dental students

Sample	Independent variable	Intervening at enamel level AND using traditional Class II preparation		Intervening at enamel level and using minimally invasive cavity preparation OR Intervening at dentin level and using traditional Class II preparation	
		OR (95% CI)^a^	*p* value	OR (95% CI)^a^	*p* value
Both dentists and dental students (n = 247)	Study group				
	Dental therapist	Reference		Reference	
	General dental practitioner	1.79 (0.59–5.46)	0.304	1.53 (0.53–4.40)	0.433
	Dental student	1.49 (0.45–4.93)	0.514	2.23 (0.73–6.76)	0.158
Only dental students (n = 76)	Sex		NA		NA
	Female	Reference		Reference	
	Male	NA		NA	
Only dentists (n = 171)	Sex		0.714		0.219
	Female	Reference		Reference	
	Male	1.36 (0.26–7.09)		2.63 (0.56–12.28)	
	Region of work		0.103		0.032
	Arkhangelsk	Reference		Reference	
	Other	3.09 (0.80–11.96)		4.15 (1.13–15.27)	
	Place of dental school graduation		NA		NA
	Arkhangelsk	Reference		Reference	
	Other	NA		NA	
	Dental practice type				
	Public	Reference		Reference	
	Private	1.04 (0.29–3.68)	0.953	1.46 (0.45–4.76)	0.533
	Both public and private	1.21 (0.32–4.65)	0.780	1.13 (0.31–4.08)	0.858
	Working experience, years		0.802		0.925
	Less than 15	Reference		Reference	
	15 and more	1.16 (0.36–3.78)		1.06 (0.34–3.24)	
	Working in urban or rural area		0.511		0.918
	Urban	Reference		Reference	
	Rural	2.08 (0.23–18.56)		1.12 (0.13–9.91)	

^a^Unadjusted odds ratios (OR) with 95% confidence intervals (95% CI) from multinomial logistic regression; the reference category of dependent variable is “Intervening at dentin level and using minimally invasive cavity preparation”

**Table 5:** Associations between restorative treatment decisions for the occlusal carious lesion and selected variables among Russian dentists and dental students

Sample	Independent variable	Intervening at enamel level AND using traditional cavity preparation		Intervening at enamel level and using minimally invasive cavity preparation OR Intervening at dentin level and using traditional preparation	
		OR (95% CI)^a^	*p* value	OR (95% CI)^a^	*p* value
Both dentists and dental students (n = 247)	Study group				
	Dental therapist	Reference		Reference	
	General dental practitioner	1.01 (0.36–2.86)	0.978	1.30 (0.67–2.55)	0.436
	Dental student	0.55 (0.18–1.63)	0.278	0.78 (0.41–1.49)	0.454
Only dental students (n = 76)	Sex		0.317		0.642
	Female	Reference		Reference	
	Male	2.70 (0.39–18.93)		1.35 (0.38–4.78)	
Only dentists (n = 171)	Sex		0.450		0.585
	Female	Reference		Reference	
	Male	1.60 (0.47–5.42)		1.27 (0.54–2.96)	
	Region of work		0.465		0.068
	Arkhangelsk	Reference		Reference	
	Other	1.48 (0.52–4.25)		1.91 (0.95–3.81)	
	Place of dental school graduation		0.766		0.234
	Arkhangelsk	Reference		Reference	
	Other	0.71 (0.08–6.76)		2.05 (0.63–6.70)	
	Dental practice type				
	Public	Reference		Reference	
	Private	0.94 (0.27–3.28)	0.926	0.66 (0.31–1.41)	0.279
	Both public and private	1.38 (0.40–4.70)	0.612	0.47 (0.20–1.10)	0.083
	Working experience, years		0.450		0.010
	Less than 15	Reference		Reference	
	15 and more	1.60 (0.47–5.42)		2.86 (1.28–6.39)	
	Working in urban or rural area		0.757		0.161
	Urban	Reference		Reference	
	Rural	1.47 (0.13–17.18)		3.07 (0.64–14.76)	

^a^Unadjusted odds ratios (OR) with 95% confidence intervals (95% CI) from multinomial logistic regression; the reference category of dependent variable is “Intervening at dentin level and using minimally invasive cavity preparation”

## Discussion

Our survey showed that the majority of Russian dentists and dental undergraduate students do not follow the MID concept when treating dental caries in permanent teeth: only a low proportion of the respondents reported that they would simultaneously intervene at dentin level and use minimally invasive cavity preparation for the presented proximal and occlusal carious lesions. Almost all participants said they would choose tooth-coloured materials for the restorations. No differences in restorative treatment decisions were observed among general dental practitioners, dental therapists, and dental students. Region of work and working experience were found to be significant factors associated with restorative treatment decisions among Russian dentists.

The present study is the first to provide information on restorative treatment decisions for proximal and occlusal carious lesions of permanent teeth among Russian dentists and dental students. We applied a questionnaire that has been widely used in international studies, and good criterion validity in relation to thresholds for initiation of restorative treatment was demonstrated. The sample of dental students was representative by sex. Nevertheless, the present findings should be interpreted with caution. Indeed, given the sampling techniques applied and the fact that the questionnaire was distributed electronically, not all dentists eligible for the survey had an equal chance of being recruited. We cannot exclude the possibility that dentists from urban areas and dentists with less than 5 years of working experience may be overrepresented in our sample. If our assumption is correct that the general population of dentists in the study area was 1490, a comparatively low number of dentists agreed to participate in the survey. There may be many explanations for this. It is possible that not all dentists from the European North-West of Russia could be reached; on the other hand, web-based studies usually have lower response rates than similar mail-based studies [11]. Indeed, the response rate in the present survey among Russian dentists (11.5%) was similar to that recorded in another web-based survey among American dentists (11.3%) [11]. Although response rate is an important indicator of study quality, there is no scientifically accepted minimum response rate for generalising study results to a target population. Moreover, non-response bias, i.e., when non-responders differ significantly from responders regarding relevant study characteristics, may be of more concern in the surveys of general populations than in those of specific groups, such as health care workers [22]. The response rate was quite high among dental students (67.9%), although the total number participating in the survey (n = 76) was still lower than the minimum sample size calculated (n = 87). Therefore, larger samples of Russian dentists and dental students and alternative recruitment methods are needed to validate our results. All of the dental students in our sample and 90% of dentists received their dental education at NSMU; therefore, it may not be possible to generalise our findings to all Russian dentists and dental students. In addition, the survey assessed self-reported information from a hypothetical situation, not the actual practices of dentists and dental students.

Based on current recommendations, the main factors used to determine intervention are the activity of the carious lesion, cleansability, and cavitation [4, 8, 23]. Restorative treatment is presently indicated for active carious lesions which are not cleansable and cavitated at dentin level. Nevertheless, cavitation cannot always be detected using tactile-visual methods, due to the specific anatomy of the occlusal surface and the presence of adjacent teeth for proximal carious lesions [4, 23]. In this case, radiographs may be used as a proxy measure to assess the likelihood of cavitation. Carious lesions, both occlusal and proximal, radiographically extending to the middle one-third of dentin or deeper are assumed to be cavitated, even if the cavitation is not clearly detectable by the naked eye, and should be managed restoratively in the majority of cases [4, 24]. In addition, restorative treatment may also be used for inactive carious lesions to restore a tooth’s form, function, and aesthetics [4].

In order to be comparable with other studies, we used a questionnaire that was developed over than 25 years ago, in which the terms “carious lesion’s activity”, “cleansability”, and “cavitation” were not clearly applied. For the proximal carious lesion, schematic radiographic grades of caries progress were presented without clinical appearance. When decisions are based on radiographs only, lesions confined to enamel are usually considered to be non-cavitated, and non-invasive/micro-invasive interventions are indicated. The cavitation status of lesions extending radiographically into the outer one-third of dentin (grade 4) is unclear. Such lesions are more likely to be non-cavitated and should be managed non-restoratively [4]; therefore grade 5 proximal caries may be considered the recommended threshold for restorative treatment [5]. Nevertheless, under certain circumstances, including patient-specific risk factors of dental caries, restorative treatment may be justified at grade 4 [4]. In our survey, we applied a less conservative approach for proximal carious lesions in the outer one-third of dentin and considered intervention at enamel level (grades 1–3) and dentin level (grades 4–6) as early and delayed, respectively. Nonetheless, the proportion of Russian dentists and dental students who reported they would intervene early (81.8% and 82.7%) was higher than that reported in the majority of studies conducted in North and South America, Western and Eastern Europe, Scandinavia, Australia, and Asia [16]. Similar results were found in Croatian (81.0%) [14] and in French (88.0%) dentists [12].

For the occlusal carious lesion, five clinical grades of caries progress were presented on photographs and the description of radiographs for every grade was given. To describe the grades, we applied the criteria employed by Espelid et al. [10], implying that grade 2 (“small cavitation clinically”) matches a lesion with cavitation at enamel level, whereas grade 3 (“moderately sized cavity”), grade 4 (“large cavity”), and grade 5 (“extensive cavity”) are lesions cavitated at dentin level. Based on this assumption, we considered intervention at enamel level (grades 1–2) and dentin level (grades 3–5) as early and delayed, respectively. Nevertheless, other criteria such as “minor loss of tooth substance” for grade 2, “moderate loss of tooth substance” for grade 3, and “considerable loss of tooth substance” for grades 4–5 have been used in other studies [11, 12], which may complicate direct comparisons with our results. Indeed, we cannot definitely conclude whether the lesions with minor, moderate, and considerable loss of tooth substance are cavitated or not. Nonetheless, grade 4 occlusal caries is considered to be closest to a gold standard threshold to initiate restorative treatment [5]. In our survey, the proportion of Russian dentists and dental students (29.4% and 26.6%) who would restore occlusal carious lesions confined to enamel was higher than that reported in the majority of studies conducted in North America, Western and Eastern Europe, Scandinavia, and Asia [16], but was lower than that found among French dentists (49.8%) in 2002 [12] and among American dentists (40.7%) in 2013 [11]. Moreover, in line with previous studies [5, 10], our survey showed that respondents who said they would intervene at the enamel level for the proximal carious lesion said the same for the occlusal carious lesion.

Along with delayed restorative treatment, the concept of MID also includes minimal cavity preparation [5–8]. Traditional cavity preparation was originally developed for the placement of dental amalgam to make a retentive cavity form, and is no longer recommended [5]. Nevertheless, in our survey, 39.8% of dentists and 34.7% of dental students said they would use traditional Class II preparation for the proximal carious lesion. The traditional preparation design was found to be the most favoured in dentists from California (54.1%) [11] and Kuwait (49.2) [15], although 68.4% of dentists in Norway [21], 59.1% in the Netherlands [5], and 54.7% in France [12] preferred to use saucer-shaped preparation for proximal carious lesions. In the present survey, only 38.6% of dentists and 41.3% of dental students said they would use the saucer-shaped technique, and 21.6% and 24.0%, respectively, would use tunnel preparation, even though saucer-shaped restorations were found to survive significantly better than tunnel restorations [25]. Moreover, in contrast with the aforementioned studies [5, 10–12, 15], a higher proportion of Russian dentists preferred a preparation including the entire occlusal fissure system. Nevertheless, in our survey, few dentists chose preparation for inlays, which usually require more invasive treatment. This finding may be explained by the fact that prosthodontists usually perform inlays in Russia.

In line with the worldwide trend [5], Russian dentists and dental students preferred resin-based composite to restore the proximal and occlusal carious lesions. Only a few dentists said they would restore the occlusal carious lesion using amalgam. Although dental amalgam in Russia is not banned officially, on the 24th of September 2014 the Russian Federation signed the Minamata Convention on Mercury, which aims to protect the environment and human health from mercury as a toxic pollutant [26]. In recent decades, the use of mercury in dental amalgam decreased from 6 tons to 0.8 tons in Russia, and amalgam fillings account for not more than 7–8% of all total dental fillings [27]. Strict government requirements for dental clinics to get permission to work with dental amalgam, and lack of safe conditions for dental personnel who perform these restorations due to mercury exposure, were reported to be the leading factors that reduced the use of amalgam in Russian dental practice [27]. This finding may also be partly explained by the increased demand of patients, who prefer to have tooth-coloured restorations [5]. None of the Russian dental students who participated in the survey said they would choose dental amalgam to restore the proximal or the occlusal carious lesion. Although dental amalgam is included in the curriculum of dental undergraduate students at NSMU, it includes only a few hours of theoretical learning.

Despite variations in treatment decisions among dental professionals from different countries, dentists still tend to use a restorative approach in the early stages of dental caries progress, and traditional cavity preparation is still quite popular [5]. As our survey has shown, Russian dentists are no exception. These findings might be partly explained by the fact that Russian dentists use outdated clinical protocols for dental caries treatment, which do not include the contemporary concept of MID. In addition, it has been hypothesised that dentists who work in areas with a high prevalence of dental caries may be more likely to use restorative treatment at early stages, instead of waiting until the lesions become less manageable [12, 28]. Indeed, a meta-analysis published in 2017 showed that dentists tend to intervene at the enamel level more often in patients at high risk for caries [16]. Given that the population of European North-West Russia has poor oral health [29], we cannot exclude that Russian dentists intervene early for this reason. Nevertheless, the progress of dental caries has been reported to be slow, and for lesions confined to enamel, more efforts should be focused on non-invasive/micro-invasive interventions followed by regular monitoring [24]. Interestingly, there were no differences in restorative treatment decisions between our dentists and dental students. This finding indicates that the curriculum in cariology at NSMU may not contain enough information on the modern treatment of dental caries and should be updated, both for dental undergraduate students and for dental professionals who attend continuous education courses at NSMU.

In some countries, a clear tendency toward delayed restorative treatment was found [5, 21]. For example, in 1983, 65.6% of Norwegian dentists preferred to use restorative treatment for proximal carious lesions confined to enamel (grades 1 and 2), but in 2009, only 7.0% of dentists reported they would intervene at these grades [21]. Moreover, using saucer-shaped preparation increased in Norway from 24.3% in 1995 to 68.4% in 2009 [21]. Researchers explained these findings by the successful adaptation of contemporary treatment strategies into dental education. The fact that older Norwegian dentists would use restorative treatment of proximal carious lesions confined to enamel more often and saucer-shaped preparation less often adds support to this explanation [21]. A similar pattern was found in our survey, as more than 15 years of working experience was significantly associated with higher odds of using only one versus both MID treatment options when restoring occlusal carious lesions. Nevertheless, no differences were found between more experienced dentists and less experienced dentists in their decision to choose both versus none of the MID treatment options. Region of work was significantly associated with restorative treatment decisions for proximal carious lesions in the present survey. Dentists working outside the Arkhangelsk Region had higher odds to use only one versus both MID treatment options. We may speculate that the proximity of the NSMU and the high number of national dental conferences and international dental forums held in the city of Arkhangelsk play an essential role in helping dentists from the Arkhangelsk Region to obtain information on contemporary approaches in dental caries management. Nonetheless, no differences in the decision to choose both versus no MID treatment options were found between dentists working within and outside of the Arkhangelsk Region.

## Conclusions

This survey showed that the majority of Russian dentists and dental undergraduate students do not follow the MID concept when treating dental caries in permanent teeth. Instead, they consider that restorative treatment of proximal and occlusal carious lesions is indicated at earlier stages than the contemporary MID concept recommends. Although Russian dentists and dental students prefer to use resin-based composite, a high proportion of the respondents said they would not use minimally invasive cavity preparation. No differences in restorative treatment decisions were found among general dental practitioners, dental therapists, and dental students. Region of work and working experience were found to be significant factors associated with restorative treatment decisions among Russian dentists. Clinical protocols on dental caries treatment and dental school curriculums should be updated to place an enhanced focus on evidence-based practice and preventive strategies. Further studies with larger samples of Russian dentists and dental students and alternative methods of recruitment are needed to validate our results.

## Data Availability

The dataset used and analysed during the current survey is available from the corresponding author on reasonable request.

## Abbreviations

MID: Minimal intervention dentistry
DMFT: Decayed missing filled teeth
NSMU: Northern State Medical University
OR: Odds ratio
CI: Confidence interval
GIC: Glass ionomer cement
